# Supplementary material for: Spontaneous hydrolysis and spurious metabolic properties of α-ketoglutarate esters
Source: Nat Commun. 2021 Aug 12;12:4905. doi: 10.1038/s41467-021-25228-9 (PMC8361106; doi:10.1038/s41467-021-25228-9)
Supplement: Supplementary file 1 — Supplementary Information [file 41467_2021_25228_MOESM1_ESM.pdf]

Supplementary Information

Spontaneous hydrolysis and spurious metabolic properties of α-ketoglutarate esters

Seth J. Parker[1,2,ƚ,*], Joel Encarnación-Rosado[1,2], Kate E. R. Hollinshead[1,2#], David M. Hollinshead[3#], Leonard J. Ash[4], Juan A. K. Rossi[2], Elaine Y. Lin[1,2], Albert S. W. Sohn[1,2], Mark R. Philips[2], Drew R. Jones[4,5], and Alec C. Kimmelman[1,2,*]

[1]Department of Radiation Oncology, New York University School of Medicine, New York, New York.
[2]Perlmutter Cancer Center, New York University School of Medicine, New York, New York.
[3]Elixir Software Ltd., Macclesfield, Cheshire, United Kingdom.
[4]Division of Advanced Research Technologies, New York University School of Medicine, New York, New York.
[5]Department of Biochemistry and Molecular Pharmacology, New York University School of Medicine, New York, New York.

ƚ *present address*: Department of Biochemistry & Molecular Biology, University of British Columbia, Vancouver, British Columbia.

# these authors contributed equally

* correspondence to seth.parker@bcchr.ca or alec.kimmelman@nyulangone.org

Alec C. Kimmelman, M.D., Ph.D.
Perlmutter Cancer Center, Department of Radiation Oncology
NYU Medical School, New York, NY 10016
alec.kimmelman@nyulangone.org

Seth J. Parker, Ph.D.
Department of Biochemistry and Molecular Biology
University of British Columbia, Vancouver, BC V6H 0B3
seth.parker@bcchr.ca

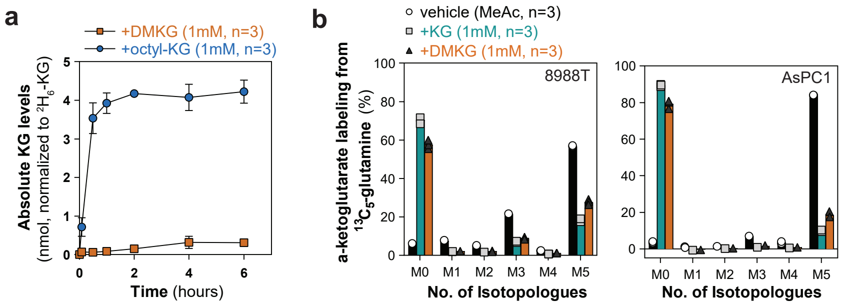
a
+DMKG (1mM, n=3)
+octyl-KG (1mM, n=3)
Absolute KG levels
(nmol, normalized to 2H6-KG)
Time (hours)
b
vehicle (MeAc, n=3)
+KG (1mM, n=3)
+DMKG (1mM, n=3)
a-ketoglutarate labeling from 13C5-glutamine (%)
8988T
AsPC1
M0
M1
M2
M3
M4
M5
No. of Isotopologues
No. of Isotopologues

Supplementary Figure 1

KG uptake and assimilation into intracellular pool. **a** Time course of absolute KG levels quantified after cell-free hydrolysis of DMKG and 1-octyl-KG over 6 hours. Data are mean ± s.d, n=3 biologically independent experiments. **b** KG mass isotopologue distribution of 8988T (left panel) and AsPC1 (right panel) cells labeled with $^{13}C_5$-glutamine and treated with either methyl acetate or 1 mM of KG or DMKG for 24 hours. Data are mean ± s.d, n=3 biologically independent samples.

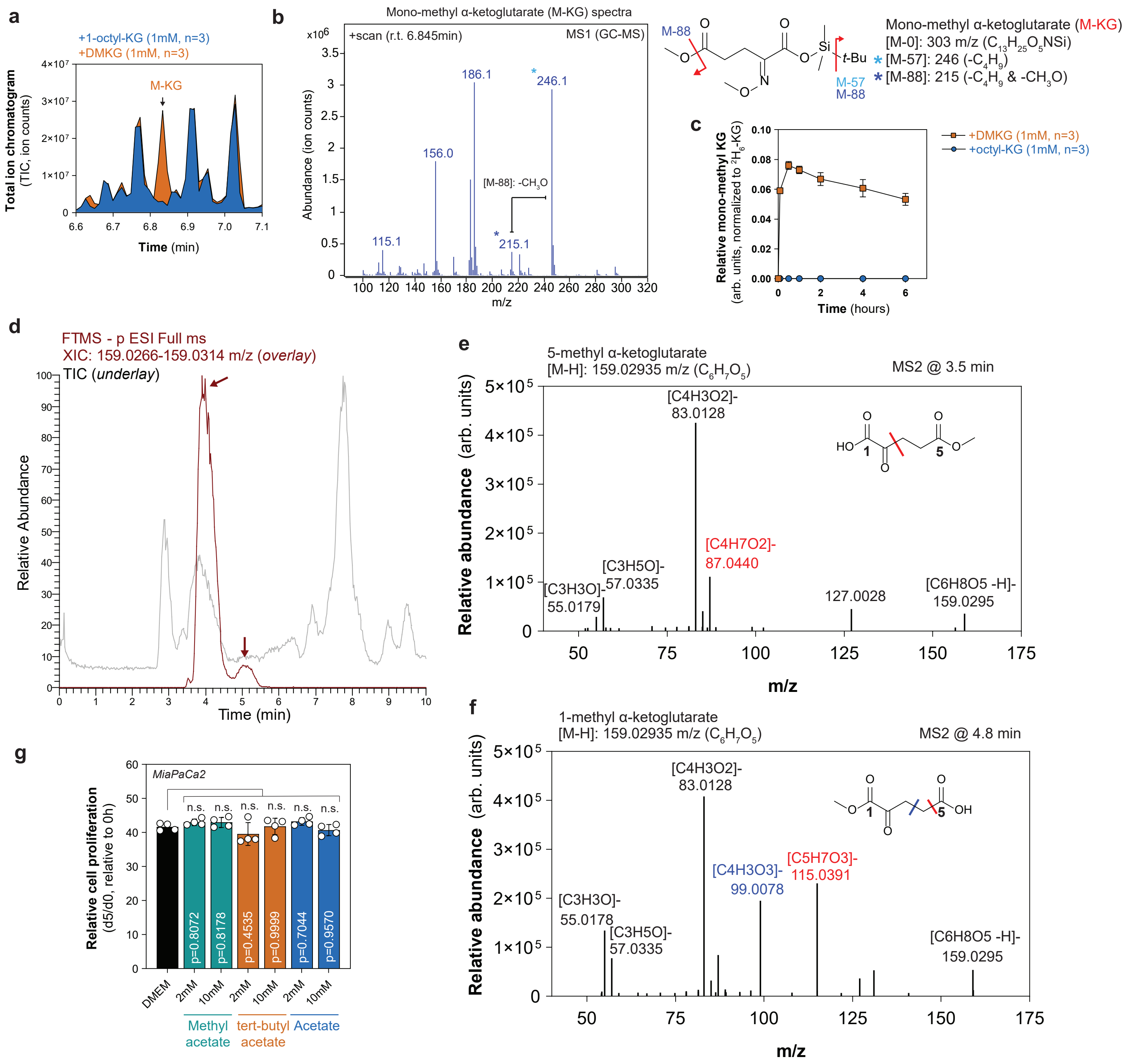

a
+1-octyl-KG (1mM, n=3)
+DMKG (1mM, n=3)
M-KG
Total ion chromatogram (TIC, ion counts)
Time (min)
b
Mono-methyl α-ketoglutarate (M-KG) spectra
+scan (r.t. 6.845min)
MS1 (GC-MS)
Abundance (ion counts)
115.1
156.0
186.1
246.1
215.1
[M-88]: -CH3O
m/z
M-88
M-57
t-Bu
Mono-methyl α-ketoglutarate (M-KG)
[M-0]: 303 m/z (C13H25O5NSi)
[M-57]: 246 (-C4H9)
[M-88]: 215 (-C4H9 & -CH3O)
c
Relative mono-methyl KG (arb. units, normalized to 2H6-KG)
+DMKG (1mM, n=3)
+octyl-KG (1mM, n=3)
Time (hours)
d
FTMS - p ESI Full ms
XIC: 159.0266-159.0314 m/z (overlay)
TIC (underlay)
Relative Abundance
Time (min)
e
5-methyl α-ketoglutarate
[M-H]: 159.02935 m/z (C6H7O5)
MS2 @ 3.5 min
Relative abundance (arb. units)
[C4H3O2]- 83.0128
[C4H7O2]- 87.0440
[C3H5O]- 57.0335
[C3H3O]- 55.0179
127.0028
[C6H8O5 -H]- 159.0295
m/z
f
1-methyl α-ketoglutarate
[M-H]: 159.02935 m/z (C6H7O5)
MS2 @ 4.8 min
Relative abundance (arb. units)
[C4H3O2]- 83.0128
[C4H3O3]- 99.0078
[C5H7O3]- 115.0391
[C3H3O]- 55.0178
[C3H5O]- 57.0335
[C6H8O5 -H]- 159.0295
m/z
g
MiaPaCa2
Relative cell proliferation (d5/d0, relative to 0h)
n.s.
p=0.8072
p=0.8178
p=0.4535
p=0.9999
p=0.7044
p=0.9570
DMEM
2mM
10mM
Methyl acetate
tert-butyl acetate
Acetate

Supplementary Figure 2

Preferential hydrolysis of DMKG to 5-methyl-KG. **a** Segment of total ion chromatogram (TIC) from 6.6 to 7.1 minutes of a representative cell-free hydrolysis GC-MS experiment of 1 mM of 1-octyl-KG (blue) or DMKG (orange) after 24 hours. Arrow indicates peak observed only in DMKG samples determined to be mono-methyl-KG (M-KG). **b** Representative GC-MS mass spectra of unique peak present in DMKG samples (*left panel*) and methoxime-tBDMS derivative fragmentation pattern (*right panel*) of mono-methyl-KG (M-KG). M-KG fragments with a main ion of 246 m/z and 215 m/z representing neutral loss of methanol and/or tert-butyl, respectively. Relative M-KG abundance in Fig. 2b was estimated from abundance of 246 m/z in hydrolysis samples. **c** Time course of relative (1/5)-methyl KG levels quantified by GC-MS after cell-free hydrolysis of 1 mM of DMKG or 1-octyl-KG over 6 hours. Data are mean ± s.d., n=3 biologically independent experiments. **d** LC-MS chromatogram from 0-10 minutes, including XIC (red, overlay) and TIC (grey, underlay), of DMKG-treated 8988T cells. Peaks depicted for XIC for 159.029 m/z ± 15 ppm unveil two peaks putatively representing 5-methyl-KG and 1-methyl-KG. Chromatogram representative of n=3 biologically independent samples. **e** Representative MS2 fragmentation spectra of parent ion 159.029 m/z at ~3.5 minutes for 5-methyl-KG depicting expected fragmentation in red. **f** Representative MS2 fragmentation spectra of parent ion 159.029 m/z at ~4.8 minutes for 1-methyl-KG depicting expected fragmentation in red and blue. **g** Relative proliferation of MiaPaCa2 cells after 5 days treatment with null-treatment (DMEM) or 2 or 10mM of methyl acetate, tert-butyl acetate, or sodium acetate. Data are mean ± s.d., n=4 biologically independent samples representative of two independent experiments; significance determined by one-way ANOVA using Dunnett's multiple comparisons test, n.s. $p>0.05$.

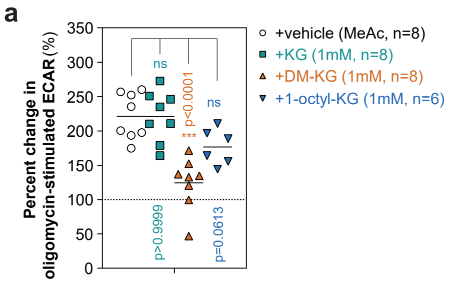
a
Percent change in oligomycin-stimulated ECAR (%)
350
300
250
200
150
100
50
0
ns
p<0.0001
***
ns
p>0.9999
p=0.0613
+vehicle (MeAc, n=8)
+KG (1mM, n=8)
+DM-KG (1mM, n=8)
+1-octyl-KG (1mM, n=6)

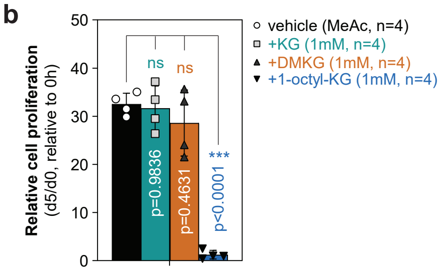
b
Relative cell proliferation
(d5/d0, relative to 0h)
50
40
30
20
10
0
ns
ns
***
p=0.9836
p=0.4631
p<0.0001
vehicle (MeAc, n=4)
+KG (1mM, n=4)
+DMKG (1mM, n=4)
+1-octyl-KG (1mM, n=4)

Supplementary Figure 3

DMKG significantly inhibits cellular glycolytic potential. **a** Percent change in oligomycin-stimulated extracellular acidification rate (ECAR) after stimulation with methyl acetate or 1 mM of KG, DMKG, or 1-octyl-KG. Following 60-minute stimulation, injection of oligomycin (oligo) was made to inhibit complex V ATP production and stimulate glycolytic flux. Data were normalized to the average of three basal ECAR values from experiment and plotted as a percentage. For each experiment, three oligomycin-stimulated datapoints were averaged and averaged across all independent experiments. Data are mean ± s.d., n= 6 (1-octyl-KG) or 8 (methyl acetate, KG, DMKG) biologically independent experiments; significance determined by one-way ANOVA using Dunnett's multiple comparisons test, n.s. $p>0.05$, *** $p<0.0001$. **b** 8988T cell proliferation after 5 days (d5) relative to initial plating density measured at treatment time (d0). Cells were treated with methyl acetate or 1 mM of KG, DMKG, or 1-octyl-KG for 5 days. Data are mean ± s.d., n= 4 biologically independent samples; Representative experiment of two independent experiments. Significance determined by one-way ANOVA using Dunnett's multiple comparisons test, n.s. $p>0.05$, *** $p<0.0001$.

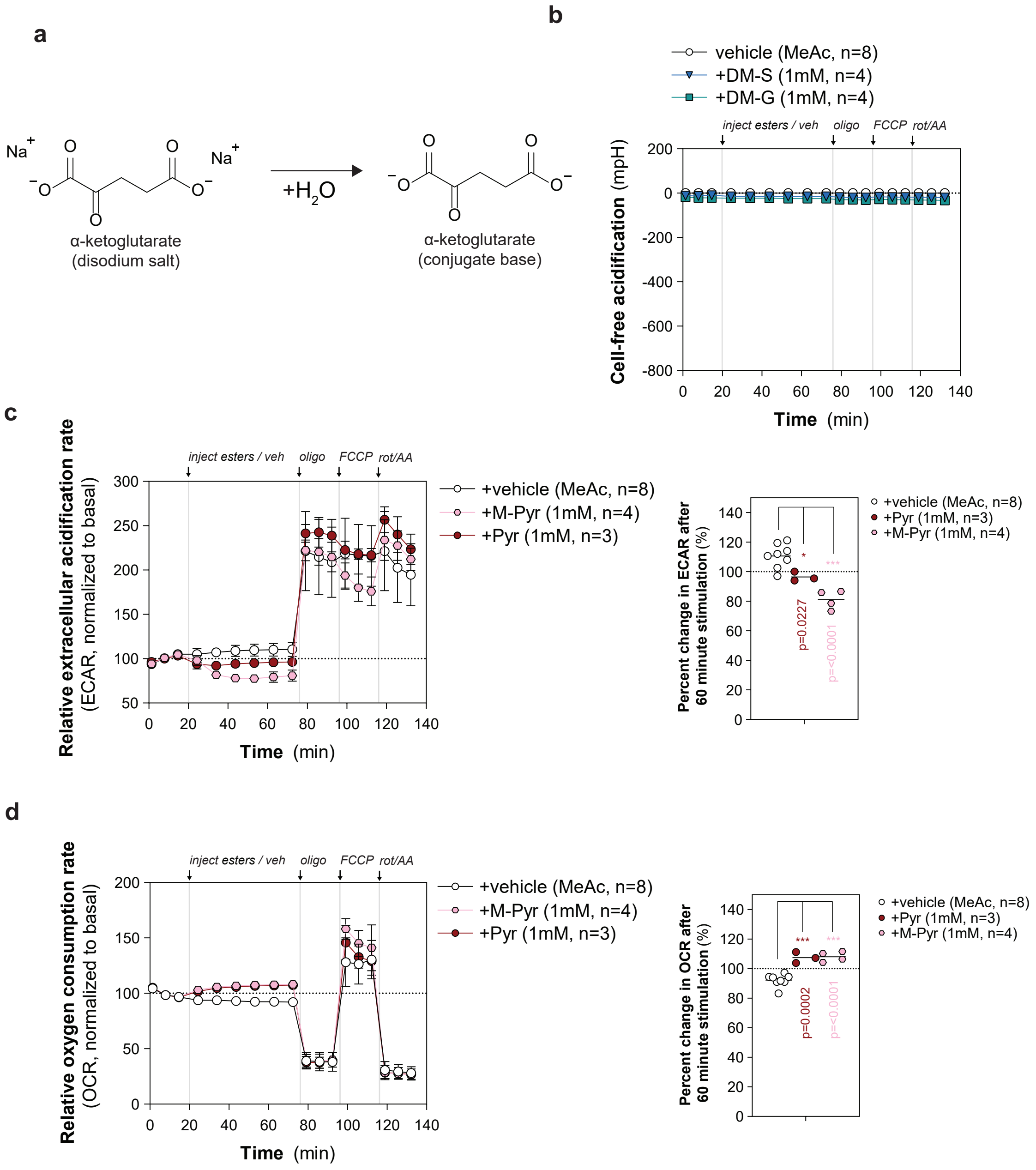
a
b
c
d
Na+
α-ketoglutarate
(disodium salt)
+H2O
α-ketoglutarate
(conjugate base)
vehicle (MeAc, n=8)
+DM-S (1mM, n=4)
+DM-G (1mM, n=4)
inject esters / veh
oligo
FCCP
rot/AA
Cell-free acidification (mpH)
Time (min)
Relative extracellular acidification rate
(ECAR, normalized to basal)
+vehicle (MeAc, n=8)
+M-Pyr (1mM, n=4)
+Pyr (1mM, n=3)
Percent change in ECAR after
60 minute stimulation (%)
+Pyr (1mM, n=3)
+M-Pyr (1mM, n=4)
p=0.0227
p=<0.0001
Relative oxygen consumption rate
(OCR, normalized to basal)
Percent change in OCR after
60 minute stimulation (%)
p=0.0002
p=<0.0001

Supplementary Figure 4

α-ketoesters hydrolysis leads to acidification and spurious impacts on metabolism. **a** Disodium salt of the conjugate base form of α-ketoglutarate does not release protons. **b** Acidification of media without cells after injection of methyl acetate or 1 mM of dimethyl-succinate (DM-S) or dimethyl-glutarate (DM-G) in units of mpH. Data are mean ± s.d of n= 4 (DM-S, DM-G) or 8 (methyl acetate) biologically independent experiments. **c** ECAR of 8988T cells upon stimulation with methyl acetate or 1 mM of sodium pyruvate (Pyr) or methyl-pyruvate (M-Pyr) (*left panel*). Percent change in ECAR of 8988T cells stimulated with methyl acetate or 1 mM of Pyr or M-Pyr after 60 minutes (*right panel*). Data are plotted as a percent relative to baseline ECAR for each biologically independent experiment prior to stimulation. Data are mean ± s.d. of n= 3 (Pyr), 4 (M-Pyr), or n=8 (methyl acetate) biologically independent experiments; significance determined (*right panel*) by one-way ANOVA using Dunnett's multiple comparisons test, * $p<0.05$, *** $p<0.0001$. **d** OCR of 8988T cells upon stimulation with methyl acetate or 1 mM of sodium pyruvate (Pyr) or methyl-pyruvate (M-Pyr) (*left panel*). Percent change in OCR of 8988T cells stimulated with methyl acetate or 1 mM of Pyr or M-Pyr after 60 minutes (*right panel*). Data are plotted as a percent relative to baseline OCR for each biologically independent experiment prior to stimulation. Data are mean ± s.d. of n= 3 (Pyr), 4 (M-Pyr), or n=8 (methyl acetate) biologically independent experiments; significance determined (*right panel*) by one-way ANOVA using Dunnett's multiple comparisons test, *** $p<0.0001$.

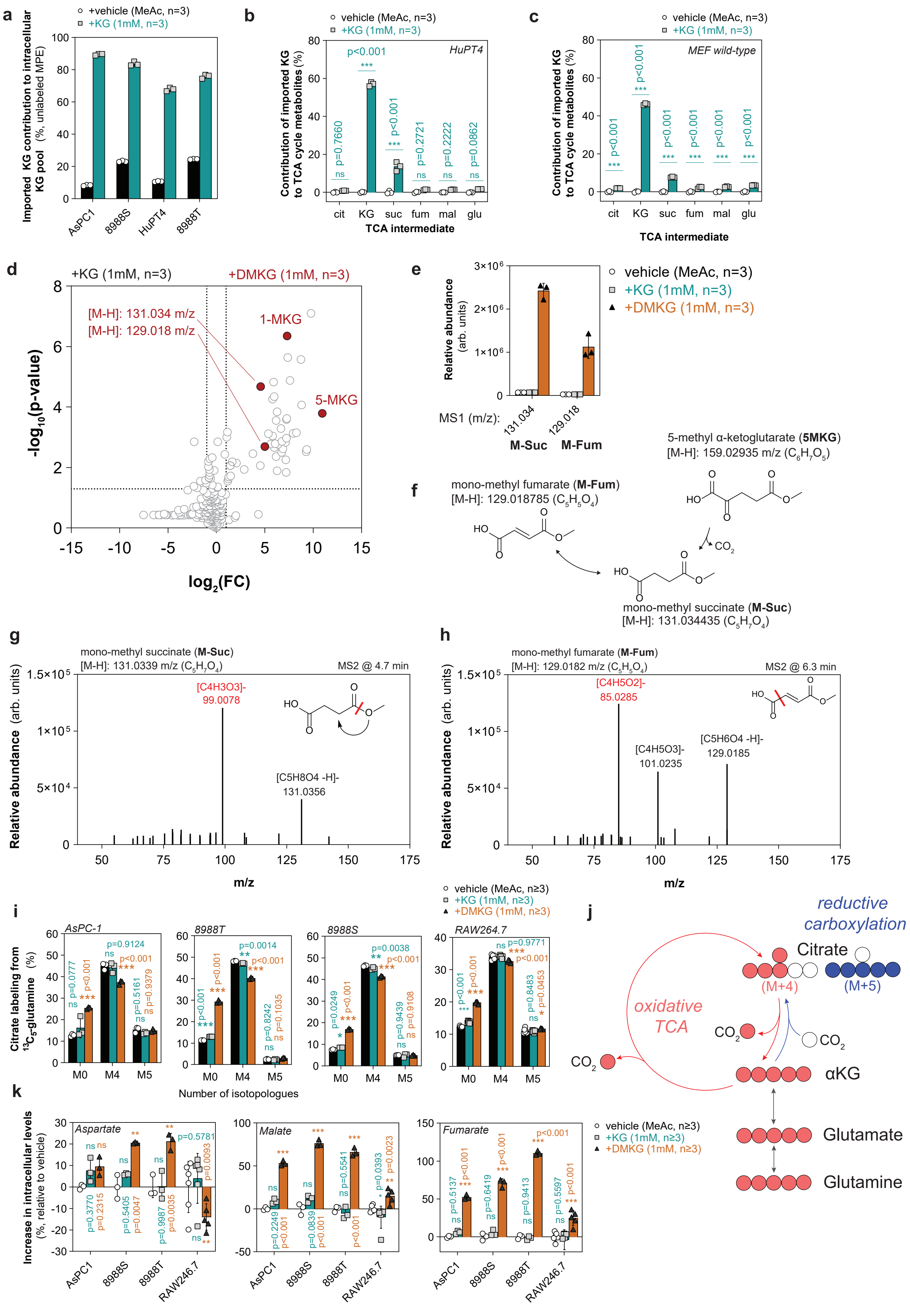

a
+vehicle (MeAc, n=3)
+KG (1mM, n=3)
Imported KG contribution to intracellular KG pool (%, unlabeled MPE)
AsPC1
8988S
HuPT4
8988T
b
vehicle (MeAc, n=3)
+KG (1mM, n=3)
HuPT4
Contribution of imported KG to TCA cycle metabolites (%)
p<0.001
p=0.7660
p<0.001
p=0.2721
p=0.2222
p=0.0862
cit
KG
suc
fum
mal
glu
TCA intermediate
c
vehicle (MeAc, n=3)
+KG (1mM, n=3)
MEF wild-type
Contribution of imported KG to TCA cycle metabolites (%)
TCA intermediate
d
+KG (1mM, n=3)
+DMKG (1mM, n=3)
[M-H]: 131.034 m/z
[M-H]: 129.018 m/z
1-MKG
5-MKG
-log10(p-value)
log2(FC)
e
vehicle (MeAc, n=3)
+KG (1mM, n=3)
+DMKG (1mM, n=3)
Relative abundance (arb. units)
MS1 (m/z):
131.034
129.018
M-Suc
M-Fum
f
5-methyl α-ketoglutarate (5MKG)
[M-H]: 159.02935 m/z (C6H7O5)
mono-methyl fumarate (M-Fum)
[M-H]: 129.018785 (C5H5O4)
CO2
mono-methyl succinate (M-Suc)
[M-H]: 131.034435 (C5H7O4)
g
mono-methyl succinate (M-Suc)
[M-H]: 131.0339 m/z (C5H7O4)
MS2 @ 4.7 min
[C4H3O3]-
99.0078
[C5H8O4 -H]-
131.0356
Relative abundance (arb. units)
m/z
h
mono-methyl fumarate (M-Fum)
[M-H]: 129.0182 m/z (C5H5O4)
MS2 @ 6.3 min
[C4H5O2]-
85.0285
[C4H5O3]-
101.0235
[C5H6O4 -H]-
129.0185
Relative abundance (arb. units)
m/z
i
vehicle (MeAc, n≥3)
+KG (1mM, n≥3)
+DMKG (1mM, n≥3)
AsPC-1
8988T
8988S
RAW264.7
Citrate labeling from 13C5-glutamine (%)
M0
M4
M5
Number of isotopologues
j
reductive carboxylation
Citrate
(M+4)
(M+5)
oxidative TCA
CO2
αKG
Glutamate
Glutamine
k
Increase in intracellular levels (%, relative to vehicle)
Aspartate
Malate
Fumarate
vehicle (MeAc, n≥3)
+KG (1mM, n≥3)
+DMKG (1mM, n≥3)
AsPC1
8988S
8988T
RAW246.7

Supplementary Figure 5

DMKG exhibits spurious metabolic effects independent of KG. **a** Contribution of imported KG to intracellular KG pool. Percent contribution calculated by measuring %(100-MPE) of labeling from $^{13}C_5$-glutamine in AsPC1, 8988S, HuPT4, or 8988T cells cultured for 24 hours with or without 1 mM unlabeled KG. Data are mean ± s.d., n=3 biologically independent samples. **b** Contribution of imported KG to mitochondrial TCA cycle intermediates, including succinate (suc), fumarate (fum), malate (mal), glutamate (glu), and citrate (cit). Percent contribution calculated by measuring the difference in %(100-MPE) from $^{13}C_5$-glutamine between KG-supplemented and control (MeAc) in HuPT4 cells cultured for 24 hours with or without 1 mM unlabeled KG. Data are mean ± s.d., n=3 biologically independent samples; significant determined by two-tailed Student's t-test corrected for multiple comparisons, n.s. p-val>0.05, *** p<0.0001. **c** Contribution of imported KG to mitochondrial TCA cycle intermediates. Percent contribution calculated by measuring the difference in %(100-MPE) from $^{13}C_5$-glutamine between KG-supplemented and control (MeAc) conditions in wild-type mouse embryonic fibroblasts (MEF) cultured for 24 hours with or without 1 mM unlabeled KG. Data are mean ± s.d., n=3 biologically independent samples; significant determined by two-tailed Student's t-test corrected for multiple comparisons, n.s. p-val>0.05, *** p<0.0001. **d** Volcano plot of untargeted features significantly altered in 8988T cells treated with methyl acetate or 1 mM of DMKG for 3 hours. Metabolomic features were considered significantly altered if $\log_2$ fold change (FC) was ≥ 1 or ≤ -1 and adjusted p-value < 0.05; significance was determined using the Wald test (DESeq2) corrected for multiple comparisons. Features with mass corresponding to predicted mass of 1-methyl-KG (1-MKG), 5-methyl-KG (5-MKG), methyl succinate (131.034 m/z, M-Suc), and methyl fumarate (129.018 m/z, M-Fum) are indicated with red circles. Data are mean ± s.d., n=3 biologically independent samples. **e** Relative levels of M-Suc and M-Fum in 8988T cells treated with methyl acetate or 1 mM of KG or DMKG for 3 hours quantified by LC-MS. Data are mean ± s.d., n=3 biologically independent samples. **f** Chemical structure and putative mechanism for metabolism of 5-methyl-KG to yield M-Suc and M-Fum. **g** Representative MS2 fragmentation spectra of parent ion 131.034 m/z at ~4.7 minutes for methyl succinate depicting expected fragmentation in red. **h** Representative MS2 fragmentation spectra of parent ion 129.018 m/z at ~6.3 minutes for methyl fumarate depicting expected fragmentation in red. **i** Citrate isotopologue labeling from $^{13}C_5$-glutamine in AsPC1, 8988T, and 8988S pancreatic cancer cells and RAW264.7 macrophages treated with methyl acetate or 1 mM of KG or DMKG for 24 hours. Unlabeled (M0) citrate and isotopologues generated from oxidative (M4) or reductive (M5) TCA cycling depicted. Data are mean ± s.d., n=3 biologically independent samples. Significance determined by two-way ANOVA using Dunnett's multiple comparisons test, n.s. p>0.05, * p<0.05, ** p<0.01, *** p<0.0001. **j** Schematic depicting glutamine utilization and isotopologue generation by oxidative TCA cycle (red) or reductive carboxylation (blue) pathways. Colored circles indicate stable-isotope labeled ^{13}C from $^{13}C_5$-glutamine; white circles indicate unlabeled, ^{12}C. **k** Relative intracellular levels of aspartate (left), malate (middle), and fumarate (right) in AsPC1, 8988S, and 8988T pancreatic cancer cells and RAW264.7 macrophages treated with methyl acetate or 1 mM of KG or DMKG for 24 hours. Signal intensity normalized to internal standard norvaline intensity and vehicle treated condition. Data are mean ± s.d., n=3 biologically independent samples. Significance determined by two-way ANOVA using Dunnett's multiple comparisons test, n.s. p>0.05, * p<0.05, ** p<0.01, *** p<0.0001.

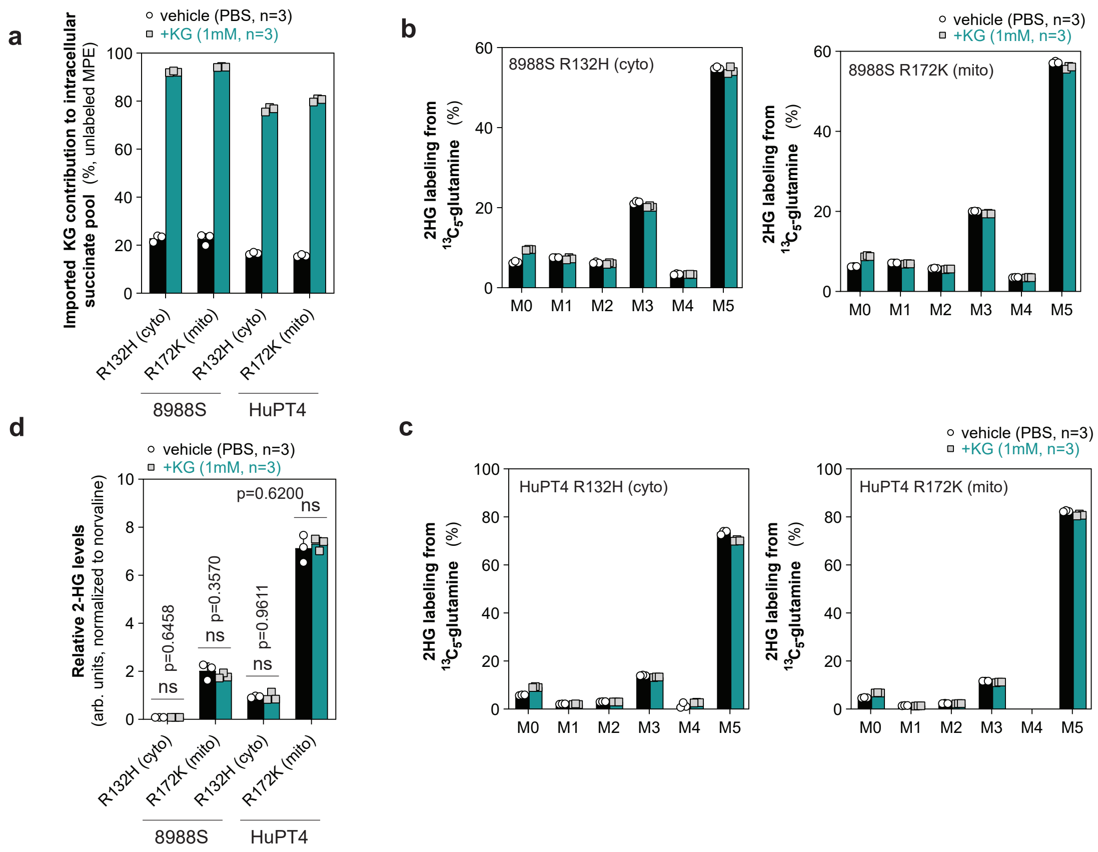
a
vehicle (PBS, n=3)
+KG (1mM, n=3)
Imported KG contribution to intracellular succinate pool (%, unlabeled MPE)
R132H (cyto)
R172K (mito)
R132H (cyto)
R172K (mito)
8988S
HuPT4
b
8988S R132H (cyto)
8988S R172K (mito)
2HG labeling from ¹³C₅-glutamine (%)
M0
M1
M2
M3
M4
M5
c
HuPT4 R132H (cyto)
HuPT4 R172K (mito)
d
Relative 2-HG levels (arb. units, normalized to norvaline)
p=0.6458
p=0.3570
p=0.9611
p=0.6200
ns

Supplementary Figure 6

Imported KG contributes minimally to mitochondrial TCA cycle metabolism. **a** Imported KG contribution to intracellular KG pool is similar between IDH1-R132H and IDH2-R172K 8988S and HuPT4 cells. Percent contribution calculated by measuring %(100-MPE) of labeling from $^{13}C_5$-glutamine with 0.5 μg/ml doxycycline with or without KG for 24 hours. Data are mean ± s.d., n=3 biologically independent samples. **b** 2-hydroxyglutarate (2-HG) mass isotopologue distribution in 8988S cells ectopically expressing dox-inducible IDH1-R132H (*left panel*) or IDH2-R172K (*right panel*) cultured with $^{13}C_5$-glutamine and 0.5 μg/ml doxycycline with or without 1 mM KG for 24 hours. Data are mean ± s.d., n=3 biologically independent samples. **c** 2-HG mass isotopologue distribution in HuPT4 cells ectopically expressing dox-inducible IDH1-R132H (*left panel*) or IDH2-R172K (*right panel*) cultured with $^{13}C_5$-glutamine and 0.5 μg/ml doxycycline with or without 1 mM KG for 24 hours. Data are mean ± s.d., n=3. **d** KG supplementation does not stimulate cytosolic or mitochondrial 2-HG production by IDH1-R132H or IDH2-R172K, respectively. Relative 2-HG levels in 8988S and HuPT4 cells treated with 0.5 μg/ml doxycycline for 24 hours to induce expression of mutant IDH1/2. Data are mean ± s.d., n=3 biologically independent samples; significance determined by two-tailed Student's t-test and corrected for multiple comparisons, n.s. $p>0.05$.
