# Supplementary material for: Spontaneous hydrolysis and spurious metabolic properties of α-ketoglutarate esters
Source: Nat Commun. 2021 Aug 12;12:4905. doi: 10.1038/s41467-021-25228-9 (PMC8361106; doi:10.1038/s41467-021-25228-9)
Supplement: Supplementary file 3 — Reporting Summary [file 41467_2021_25228_MOESM3_ESM.pdf]

nature portfolio

Corresponding author(s): Kimmelman, A.C. and Parker, S.J.

Last updated by author(s): Jul 23, 2021

Reporting Summary

Nature Portfolio wishes to improve the reproducibility of the work that we publish. This form provides structure for consistency and transparency in reporting. For further information on Nature Portfolio policies, see our Editorial Policies and the Editorial Policy Checklist.

Statistics

For all statistical analyses, confirm that the following items are present in the figure legend, table legend, main text, or Methods section.

n/a	Confirmed	
☐	☒	The exact sample size (*n*) for each experimental group/condition, given as a discrete number and unit of measurement
☐	☒	A statement on whether measurements were taken from distinct samples or whether the same sample was measured repeatedly
☐	☒	The statistical test(s) used AND whether they are one- or two-sided<br>*Only common tests should be described solely by name; describe more complex techniques in the Methods section.*
☐	☒	A description of all covariates tested
☐	☒	A description of any assumptions or corrections, such as tests of normality and adjustment for multiple comparisons
☐	☒	A full description of the statistical parameters including central tendency (e.g. means) or other basic estimates (e.g. regression coefficient) AND variation (e.g. standard deviation) or associated estimates of uncertainty (e.g. confidence intervals)
☐	☒	For null hypothesis testing, the test statistic (e.g. *F*, *t*, *r*) with confidence intervals, effect sizes, degrees of freedom and *P* value noted<br>*Give P values as exact values whenever suitable.*
☒	☐	For Bayesian analysis, information on the choice of priors and Markov chain Monte Carlo settings
☒	☐	For hierarchical and complex designs, identification of the appropriate level for tests and full reporting of outcomes
☒	☐	Estimates of effect sizes (e.g. Cohen's *d*, Pearson's *r*), indicating how they were calculated

Our web collection on statistics for biologists contains articles on many of the points above.

Software and code

Policy information about availability of computer code

Data collection	No software was used.
Data analysis	- Metabolyze is available at https://github.com/DrewRJones/Metabolyze_Public.git (Accessed on July 10, 2021) under MIT license.<br>- GC-MS analysis script is available at https://github.com/Sethjparker/IntegrateNetCDF_WithCorrect (Accessed on July 10, 2021) under MIT license.<br>- METLIN (2017) is available from https://www.sisweb.com/software/ms/wiley-metlin.htm (Accessed on July 10, 2021).<br>- Python v3.6.5<br>- Python SciPy v1.1.0<br>- R v3.5.1<br>- Matlab R2020a<br>- Graphpad Prism v9<br>- DESeq2 v1.24.0<br>- Adobe Illustrator 2021<br>- Microsoft Office Professional Plus 2019

For manuscripts utilizing custom algorithms or software that are central to the research but not yet described in published literature, software must be made available to editors and reviewers. We strongly encourage code deposition in a community repository (e.g. GitHub). See the Nature Portfolio guidelines for submitting code & software for further information.

Data

Policy information about availability of data

All manuscripts must include a data availability statement. This statement should provide the following information, where applicable:

- Accession codes, unique identifiers, or web links for publicly available datasets
- A description of any restrictions on data availability
- For clinical datasets or third party data, please ensure that the statement adheres to our policy

The LC-MS data generated in this study have been deposited in the National Metabolomics Data Repository (https://www.metabolomicsworkbench.org/) under study ID ST001860. The processed GC-MS data generated in this study are provided in the Source Data file. METLIN (2017) is available from https://www.sisweb.com/software/ms/wiley-metlin.htm (Accessed on July 10, 2021). Other data associated with this study are available from the corresponding author(s) upon reasonable request.

Field-specific reporting

Please select the one below that is the best fit for your research. If you are not sure, read the appropriate sections before making your selection.

☒ Life sciences ☐ Behavioural & social sciences ☐ Ecological, evolutionary & environmental sciences

For a reference copy of the document with all sections, see nature.com/documents/nr-reporting-summary-flat.pdf

Life sciences study design

All studies must disclose on these points even when the disclosure is negative.

Sample size	A sample size of three biologically independent samples was used for each cellular stable-isotope tracing or cell-free hydrolysis experiment. No technical sampling was performed on biologically independent samples. This number of biologically independent replicates captures the biological variability observed from previous uptake, transport, and metabolism studies published by the authors (PMID: 32341021, 28034771, 24882210, 24755473). For cell-free Seahorse experiments, at least 2 technical replicate wells and at least 3 independent experiments were included for each experimental condition to control. For in-cell Seahorse assays, at least 6 technical replicate wells and at least 3 independent experiments were included per experimental condition. These numbers capture the technical variability of Seahorse OCR/ECAR/pH assays observed in previous studies published by the authors (PMID: 32341021, 33027658, 31827279, 28034771, 24755473).
Data exclusions	No data were excluded from analysis.
Replication	To replicate our findings, we conducted multiple independent and orthogonal experiments and/or used multiple different cell lines. The cell lines used and number of independent experiments are indicated in associated figure legends for each result. For independent experiments with multiple biologically independent replicates, replicates were averaged for each independent experiment and statistical analysis was performed on the mean and variances of all independent experimental data.
Randomization	For in vitro cell-based assays, all cell plating was randomized between treated and untreated/vehicle treated conditions. Treatment for Seahorse OCR/ECAR experiments was not randomized to maintain consistent well assignments between independent experiments; however, cell plating for these assays was randomized. To control for unexpected covariates for Seahorse OCR/ECAR experiments, multiple independent experiments were conducted. Sample collection of cell-free hydrolysis was randomized between conditions. Sample acquisition by GC-MS or LC-MS was randomized to control for instrument bias and unexpected covariates.
Blinding	Studies were not blinded because experimental and surrogate counting wells needed to be assigned and treated similarly during treatment. Cell-free hydrolysis was partially blinded; samples were assigned letter codes that were documented ahead of the experiment and sample collection was randomized between letter coded sample vials.

Reporting for specific materials, systems and methods

We require information from authors about some types of materials, experimental systems and methods used in many studies. Here, indicate whether each material, system or method listed is relevant to your study. If you are not sure if a list item applies to your research, read the appropriate section before selecting a response.

Materials & experimental systems

n/a	Involved in the study	
☒	☐	Antibodies
☐	☒	Eukaryotic cell lines
☒	☐	Palaeontology and archaeology
☒	☐	Animals and other organisms
☒	☐	Human research participants
☒	☐	Clinical data
☒	☐	Dual use research of concern

Methods

n/a	Involved in the study	
☒	☐	ChIP-seq
☒	☐	Flow cytometry
☒	☐	MRI-based neuroimaging

Eukaryotic cell lines

Policy information about cell lines

Cell line source(s)	As detailed in the methods section: The cell lines AsPC1, 8988S, HuPT4, PANC1, 8988T, MiaPaCa2, DanG, and RAW246.7 were obtained from ATCC or the DMSZ. Wild-type MEFs were a gift from the DePinho Laboratory (The University of Texas MD Anderson Cancer Center) and were generated as previously described (PMID: 11544531). 293T cells were a gift from William Hahn (Dana-Farber Cancer Institute).
Authentication	As detailed in the methods section: All cell lines were authenticated by STR DNA fingerprinting within the last two years, and a central cell bank was maintained containing authenticated cell lines.
Mycoplasma contamination	As detailed in the methods section: At least once prior to all experiments, cell lines were verified to be mycoplasma free by PCR.
Commonly misidentified lines (See ICLAC register)	Commonly misidentified cell lines were not used in this study.
